# Superhydrophobic Modification of Biomass Cuttlebone Applied to Oil Spill Remediation

**DOI:** 10.3390/ma15134401

**Published:** 2022-06-22

**Authors:** Junfei Xu, Pengchao Che, Hailong Zhang, Yuliang Zhang, Jun Wu, Weiqi Li, Jizhong He, Zhihui Ma, Tengfei Li, Yunyuan Dong, Jianping Yu, Ruiping Tong

**Affiliations:** 1 Key Laboratory of Air-Driven Equipment of Zhejiang Province, College of Mechanical Engineering, Quzhou University, Quzhou 324000, China; chepengchaomail@163.com (P.C.); zhl19980521@163.com (H.Z.); zhang002@sina.com (Y.Z.); wujun4182@126.com (J.W.); liweiqi2714941535@163.com (W.L.); h18966409133@163.com (J.H.); ma15135236443@126.com (Z.M.); leetengfly@126.com (T.L.); yujianping@zju.edu.cn (J.Y.); 2 College of Chemical & Materials Engineering, Quzhou University, Quzhou 324000, China; dyy20191201@163.com

**Keywords:** seafood byproduct, cuttlebone, biomass, superhydrophobicity, oil spill remediation

## Abstract

The spills of crude oil and other organic chemicals are common around the world, resulting in severe damage to the environment and ecosystem. Therefore, developing low-cost and eco-friendly absorption material is in urgent need. In this study, we report a superhydrophobic and oleophilic porous material using biomass cuttlebone as the scaffold. A layer of polydopamine is grafted on the cuttlebone as the adhesion layer between the cuttlebone and the superhydrophobic coating. The in situ grown silica micro/nanoparticles on top of the adhesion layer provide the anchoring spots for grafting the fluorinated hydrocarbon and a rough topography for realizing superhydrophobicity. The static water contact angle of the superhydrophobic cuttlebone reaches 152°, and its oil contact angle is ~0°. The excellent oil–water separation efficiency of the prepared superhydrophobic cuttlebone is demonstrated using high-density oil/water mixtures and low-density oil/water mixtures.

## 1. Introduction

Marine pollutants, such as untreated human sewage, spilled oil, and industrial chemical waste, have caused serious environmental and ecological damage. Among them, spilled oil is the most harmful and destructive marine pollutant [1,2,3,4,5,6]. The ever-increasing demand for crude oil has resulted in many oil spills over the years, such as Odyssey oil spill, ABT Summer oil spill, and the Deepwater Horizon oil spill. These big oil spills could cause severe and extensive damage to the marine and near-shore environment and ecosystem for decades to come. Therefore, developing affordable oil spill remediation is in urgent need. Among various oil spill remediation technologies, the superhydrophobic absorbing strategy is of great interest. The development of highly efficient and low-cost superhydrophobic and lipophilic materials is promising for oil spill management [7].

Absorbing materials such as polymers, natural fibers, and inorganic powders have been utilized to absorb oil in oil/water mixtures [8,9,10,11,12,13,14]. Ruan et al. synthesized a flame-retardant superhydrophobic sponge using melamine and formaldehyde, and the sponge showed excellent oil absorption capability and flame retardancy [1]. Cheng et al. prepared a superhydrophobic polyurethane sponge for efficient separation of immiscible oil/water mixtures and emulsions [15]. Duan et al. reported a superhydrophobic chitin-based sponge as an efficient oil absorber [16]. Arbatan et al. synthesized a superhydrophobic calcium carbonate powder using a simple, low-cost preparation method, and it showed high efficiency in separating oil/water mixtures [17]. Although fruitful results have been achieved in developing materials with superhydrophobic and lipophilic properties, practical applications of these materials in cleaning up oil spills are still hindered by their manufacturing technologies, cost, and environmental impacts.

Cuttlefish as marine mollusks have a unique internal shell skeleton, i.e., cuttlebone [18,19,20,21]. It has a rigid surface and a porous honeycomb-like internal structure. With high porosity (~93%) and excellent mechanical properties, cuttlebone has great potential for fabrication of new materials [18,20]. Compared to cellulose aerogel [22,23,24,25,26,27,28] and chitin aerogel [16,29], and chitin/cellulose aerogel [30], the high porosity, ultra-low density, high permeability, and high robustness of cuttlebone make it a great candidate for high-performance adsorption materials [31,32,33].

Superhydrophobic and oleophilic materials have gained tremendous research interest for oil–water separation applications [34,35,36,37,38]. Herein we report a novel strategy for the preparation of a superhydrophobic and oleophilic material, using low-cost and easily available cuttlebone as the scaffold. A simple and convenient three-step method is used to convert the surface of cuttlebone from superhydrophilicity to superhydrophobicity. A thin layer of polydopamine is first grafted onto the surface of the cuttlebone by immersing the samples in an aqueous solution of dopamine. Then, a layer of silica micro/nanoparticles is grown on the polydopamine-modified cuttlebone. Finally, the surface of the sample is modified with a fluoroalkyl silane. The as-obtained superhydrophobic cuttlebone exhibits excellent oil absorption performance. The morphology, internal structure, and physical and chemical properties of the untreated cuttlebone and the superhydrophobic cuttlebone were characterized. Our work provides a new strategy for minimizing the environmental and ecological impacts of oil spills.

## 2. Materials and Methods

### 2.1. Materials

Cuttlebone was provided by BoZhou Sea Medicines Procurement Co., Ltd., Bozhou, China. Dichloromethane, acetone, n-hexane, anhydrous ethanol, ammonia solution, tetraethyl silicate, heptadecafluoro-1,1,2,2,-tetrahydrodecyl dimethylchlorosilane, and dopamine hydrochloride were purchased from Macklin Biochemical Co., Ltd., Shanghai, China. Diesel oil was purchased from Sinopec gas station. Edible blend oil was purchased from Shandong Luhua Group Co., Ltd., Yantai, China.

### 2.2. Preparation of Polydopamine-Modified Cuttlebone

The hard shell of the cuttlebone was removed, and then the remaining cuttlebone was cut into cylinders with a diameter of 2 cm and a height of 2 cm. Next, the cuttlebone cylinders were immersed in acetone for 1 h and deionized water for 1 h to wash away dust and impurities. The cuttlebone cylinders were then dried in an oven at 100 °C for 2 h. After that, the dry cuttlebone cylinders were dipped into an aqueous solution of dopamine (in 10 mM Tris-buffer), leading to the spontaneous deposition of a thin layer of polydopamine over the entire scaffold. After 6 h of soaking, the yellowish-brown cuttlebone cylinders were washed with deionized water 3 times. Finally, the cuttlebone cylinders were dried in an oven at 60 °C to obtain polydopamine-modified cuttlebone.

### 2.3. Preparation of Superhydrophobic and Oleophilic Cuttlebone

**Figure 1:**
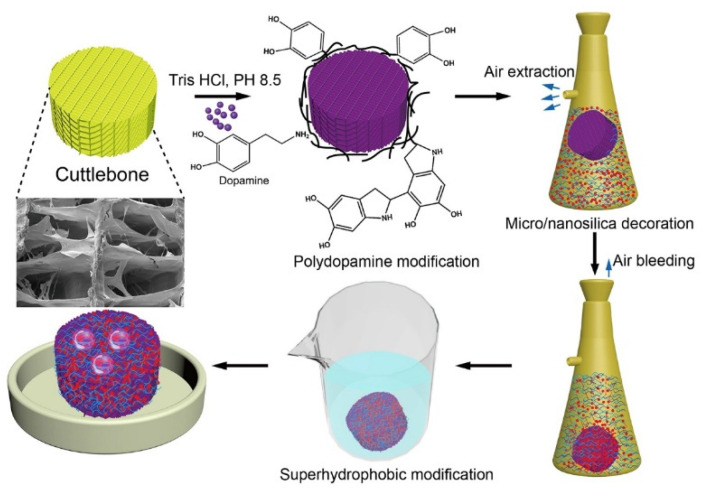
Schematic of surface modification of superhydrophobic and oleophilic cuttlebone cylinders.

A total of 5 mL of 30% ammonia solution was combined with 65 mL of anhydrous ethanol, and the mixture was mechanically stirred for 10 min to form solution A. 5 mL of TEOS was combined with 25 mL of anhydrous ethanol, and the mixture was mechanically stirred for 20 min to form solution B. 30 mL of solution B was added dropwise into 70 mL of solution A to form solution C. Then the polydopamine-modified cuttlebone cylinders were placed into a filter flask, followed by adding solution C. After that, the filter flask was pumped down to remove the air trapped in the cuttlebone cylinders. When the cuttlebone cylinders stopped bubbling, the flask was unplugged, and the cuttlebone cylinders sank to the bottom of the flask as their pores were displaced by solution C. After being dried at 80 °C, the micro/nanosilica-decorated cuttlebone cylinders were obtained. Finally, the micro/nanosilica-decorated cuttlebone cylinders were immersed in 300 mL of 0.1% trichlorooctadecyl silane solution for 3 h. After being dried at 100 °C, the superhydrophobic cuttlebone was obtained. The schematic of the preparation process is shown in Figure 1.

### 2.4. Characterization

#### 2.4.1. Scanning Electron Microscopy (SEM) Analysis

A field emission scanning electron microscope (Merlin, Zeiss, Germany) was used to investigate the morphology and internal microstructure of cuttlebone samples. The working voltage was 1.5 kV and the current was 1 CVA. The samples were prepared using the following method: The dried cuttlebone samples were cut into 0.5 cm × 0.5 cm × 0.5 cm slices and then attached to the sample holders using conductive glue, followed by sputtering a thin layer of gold. 

#### 2.4.2. Fourier Infrared Spectroscopy (FTIR) Analysis

Chemical structural and composition changes in the untreated cuttlebone, the polydopamine-modified cuttlebone, and the superhydrophobic cuttlebone were analyzed by FTIR (VERTEX 70, Bruker, Germany). Before the test, the samples were completely dried to eliminate the interference of the strong OH absorption peak. Then, each dried sample (1 mg) was mixed with spectral pure KBr (100 mg) and ground into fine powder. Finally, the mixture was pressed to form a thin film for FTIR testing.

#### 2.4.3. X-ray Photoelectron Spectroscopy (XPS) Analysis

An AXIS Ultra DLD X-ray photoelectron spectrometer (K-Alpha+, Thermo Fisher Scientific, Waltham, MA, USA) was used to analyze the chemical composition and elemental content and valence on the surface of the untreated cuttlebone, the polydopamine-modified cuttlebone, and the superhydrophobic cuttlebone. Before the analysis, the samples were dried and grounded into powder. The XPS spectra of C, O, N, F, and Si were analyzed.

#### 2.4.4. Static Contact Angle (CA) Analysis

The contact angles of the untreated cuttlebone and the superhydrophobic cuttlebone were determined by the static contact angle analyzer (Zetasizer Nano, Malvern, UK). The analysis was carried out by placing a sample horizontally on the sample stage and then dropping a drop of 3 μL of distilled water onto the sample surface. The measurement of the CA between the water droplet and the sample surface was repeated three times at different positions of each sample.

#### 2.4.5. Oil Absorption Capacity Analysis

A superhydrophobic cuttlebone sample was immersed into various oil/organic solvents for 10 min. After that, the sample was removed from the oil/organic solvent, and its excess surface oil/organic solvent was drained. Next, the sample was weighted. This test was repeated 3 times, and the averaged weight was used for the following analysis. The absorption capacity, *a*, is defined as

(1) $$a=\frac{m_{t}-m_{i}}{m_{i}}$$

where *m_i_* and *m_t_* are the weight of the superhydrophobic cuttlebone sample before and after oil absorption.

#### 2.4.6. Oil–Water Separation Efficiency Analysis

A water–oil mixture was prepared by combining 1 g of oil/organic solvent (containing 3% of Sudan III) and 4 g of deionized water in a beaker. Then, a superhydrophobic cuttlebone sample was placed in the beaker to absorb the oil. The test was repeated 3 times and the average weight of the oil–water mixture before and after the test was used for the following analysis. The oil–water separation efficiency, *e*, is defined as follows:

(2) $$e=\frac{m_{z}-m_{g}}{m_{o}}$$

where *m_z_*, *m_g_*, and *m_o_* are the average weight of the oil–water mixture before the test, the average weight of the oil–water mixture after the test, and the weight of the oil used for preparing the oil–water mixture, respectively.

## 3. Results and Discussion

### 3.1. Morphology and Composition Characterization

**Figure 2:**
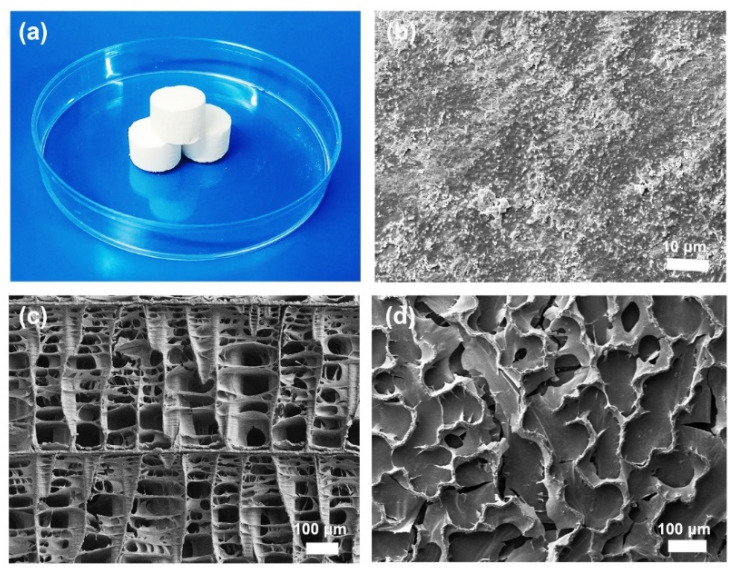
(**a**) Untreated cuttlebone cylinders (2 cm in height and 2 cm in diameter), (**b**) SEM image of the internal surface of an untreated cuttlebone sample, (**c**) SEM image of the cross section of an untreated cuttlebone sample cut longitudinally, (**d**) SEM image of the cross section of an untreated cuttlebone sample cut transversely.

Cuttlebone is a porous lamellar septa structure covered by a hard calcium carbonate shell. When cuttlebone is cut longitudinally, the cross section presents a highly ordered structure consisting of walls and septa (Figure 2c). The walls are connected by ordered parallel sheets, which consist of chitin and calcium carbonate. When cuttlebone is cut transversely, the cross section shows that the walls are in wavier profiles forming interconnected “S” shaped channels (Figure 2d). The SEM analysis shows that the surface of the “S” shaped channel has uniformly distributed nano-sized calcium carbonate particles (Figure 2b), which are adsorbed through electrostatic force. This structure has good mechanical strength, allowing the cuttlefish to resist high pressure up to 200 m below water, and the “S” shaped channel in the cuttlebone provides cuttlefish with an efficient way to maintain neutral buoyancy by changing the liquid-to-air ratio. Therefore, with its unique structure, cuttlebone has great potential in the development of superhydrophobic and lipophilic materials for oil–water separation. In this study, the hard shell of the cuttlebone samples was first removed, and then the remaining porous structure was cut into cylinders (2 cm in height and 2 cm in diameter, as shown in Figure 2a) for the following manufacturing processes.

**Figure 3:**
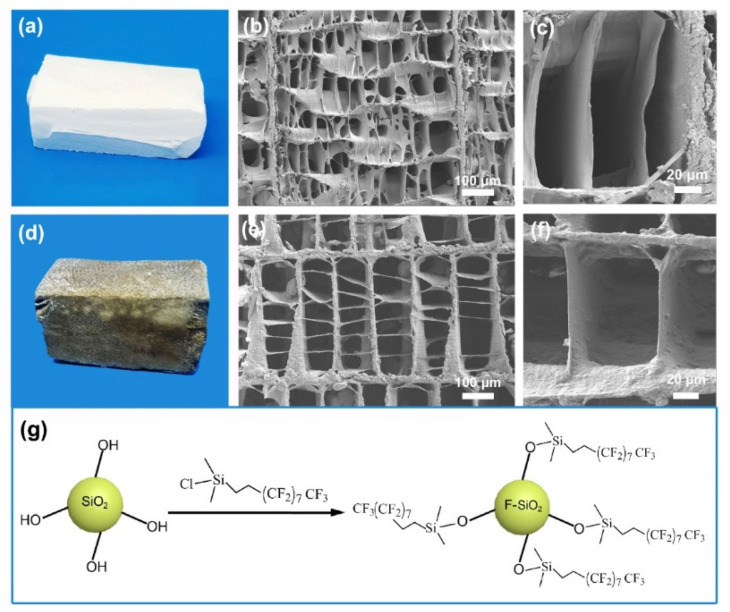
(**a**) Photograph of an untreated cuttlebone sample, (**b**) low-magnification SEM image of the cross-section of an untreated cuttlebone sample, (**c**) high-magnification SEM image of the lamellar structure of an untreated cuttlebone sample, (**d**) photograph of a superhydrophobic cuttlebone sample, (**e**) low-magnification SEM image of the cross-section of a superhydrophobic cuttlebone sample, (**f**) high-magnification SEM image of the lamellar structure of a superhydrophobic cuttlebone sample, (**g**) the reaction scheme of preparing fluorinated silica particles.

A cuttlebone can be easily processed into various shapes as required (Figure 3a). Figure 3b shows that the untreated cuttlebone has a three-dimensional highly porous structure, indicating a high absorption capacity. The surface of the walls and septa of the untreated cuttlebone is smooth, as shown in Figure 3c. For oil–water separation, natural cuttlebone requires to be converted from hydrophilic to superhydrophobic. Therefore, rapid superhydrophobic modification of cuttlebone is the key to this study.

To improve the stability of the superhydrophobic layer, a layer of polydopamine was first grafted on the surface of the external and internal structures of the cuttlebone samples. The polydopamine layer acts as an adhesion mediator between the superhydrophobic layer and cuttlebone. With a large number of micro/nano-sized pores, the inner structures of the polydopamine-modified cuttlebone cannot be fully wetted when placed in an aqueous solution. Hence, a vacuuming and releasing method (see method section for details) was adopted to rapidly wet the polydopamine-modified cuttlebone samples with solution C for the in-situ growth of silica micro/nanoparticles on the surface of the cuttlebone samples. After that, the samples were dried, and the in-situ grown silica particles on the surface of the cuttlebone samples were further reacted with fluorosilane to form a superhydrophobic layer (Figure 3g). As shown in Figure 3a,d, the color of the superhydrophobic cuttlebone changes from white to yellowish-brown, which is mainly due to the presence of the polydopamine layer. The SEM image of the superhydrophobic cuttlebone shows that the layered porous structure of the untreated cuttlebone is preserved (Figure 3e), indicating that polydopamine and superhydrophobic modification have little influence on the original structure of the cuttlebone. The high-resolution SEM image (Figure 3f) shows that the surface of the superhydrophobic cuttlebone sample is covered with a layer of silica micro/nanoparticles, and the walls and septa become thicker and rougher compared to that of the untreated cuttlebone.

**Figure 4:**
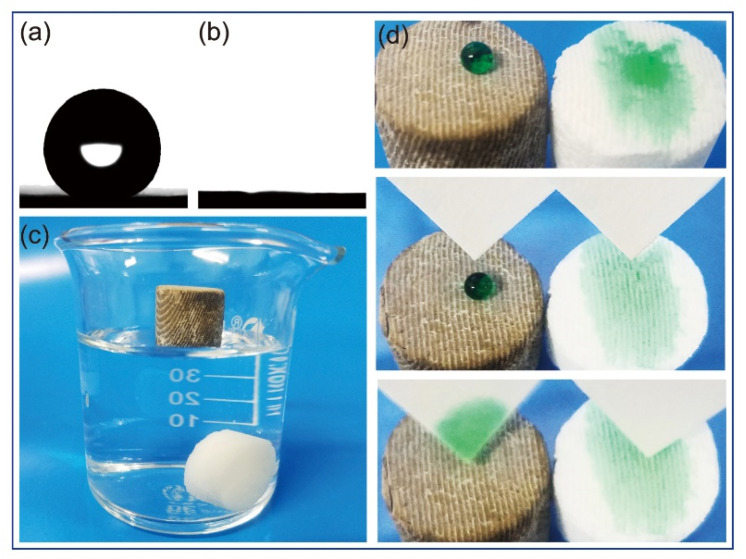
(**a**) Photograph of a water droplet on the surface of a superhydrophobic cuttlebone sample, (**b**) photograph of a water droplet on the surface of an untreated cuttlebone sample, (**c**) photograph of an untreated cuttlebone sample and a superhydrophobic cuttlebone sample placed in a beaker with water, (**d**) snapshots of the water-repelling test of the samples.

The wetting properties of the samples were characterized by measuring the static contact angle using a contact angle analyzer. As shown in Figure 4d, a water droplet was dropped on the surface of a superhydrophobic cuttlebone sample and an untreated cuttlebone sample. As the untreated cuttlebone is hydrophilic, the water droplet was absorbed by the sample, resulting in a static water contact angle of ~0° (Figure 4b). In contrast, the water droplet on the superhydrophobic cuttlebone sample formed a sphere with a static water contact angle of ~152° (Figure 4a). To demonstrate the water-repelling property of the superhydrophobic cuttlebone sample, filter papers were used to remove the water droplets dropped on the surfaces of the superhydrophobic cuttlebone sample and the untreated cuttlebone sample. As shown in Figure 3d, the water droplet on the superhydrophobic cuttlebone can be fully removed without leaving any residue, while the water drop on the untreated cuttlebone has already been absorbed and cannot be removed by the filter paper.

To demonstrate the oil–water separation performance of the superhydrophobic cuttlebone, a vacuuming and releasing treatment of superhydrophobic and untreated cuttlebone samples was first carried out in a filter flask with water. Both samples were then placed in a beaker with water (Figure 4c). The untreated cuttlebone sample sinks to the bottom, while the superhydrophobic cuttlebone sample floats on the surface.

**Figure 5:**
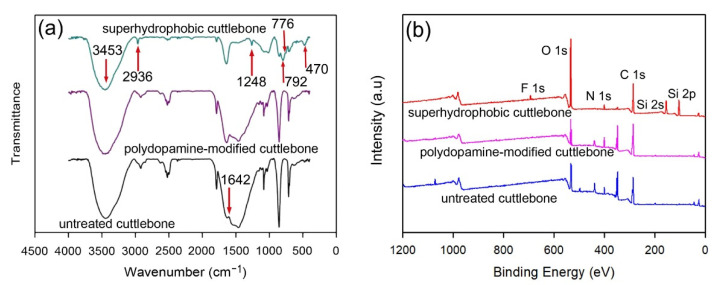
FTIR spectra (**a**) and XPS spectra (**b**) of the untreated cuttlebone, the polydopamine-modified cuttlebone, and the superhydrophobic cuttlebone.

The chemical compositions of the cuttlebone samples before and after modification were investigated by FTIR. The results are shown in Figure 5a. The spectra of the untreated cuttlebone, the polydopamine-modified cuttlebone, and the superhydrophobic cuttlebone exhibit a strong absorption peak at 3453 cm^−1^, which could be assigned to the stretching vibration of the -OH group. The characteristic absorption peak at 2936 cm^−1^ corresponds to the stretching vibrations of C-H_4_, and the peak at 1642 cm^−1^ corresponds to the N-H stretching [39]. The weak peak at 1642 cm^−1^ of the untreated cuttlebone is due to its low chitin content. As polydopamine contains N-H bonds, the polydopamine-treated cuttlebone shows a strong peak at 1642 cm^−1^ compared with the untreated cuttlebone. The absorption peak at 3453 cm^−1^ in the spectrum of the polydopamine-modified cuttlebone could be assigned to the superimposed stretching vibration of the N-H bonds and O-H bonds. In the spectrum of the superhydrophobic cuttlebone, the adsorption peaks at 1248 cm^−1^ and 776 cm^−1^ could be ascribed to the stretching vibrations of the C-F bonds and Si-O-Si bonds, and the peak at 470 cm^−1^ could be ascribed to the stretching vibrations of the Si-O bond in SiO_2_ [29]. These results indicate that the surface modification using polydopamine and fluorinated silica is successful. The successful grafting of the hydrophobic molecules on the surface of the superhydrophobic cuttlebone was confirmed by XPS. The XPS spectra in Figure 5b reveal the presence of the elements of silicon and fluorine on the surface of the superhydrophobic cuttlebone, showing the characteristic peaks at 105.3 eV (Si2p), 156.2 eV (Si2s), and 692.6 eV (F1s) [40].

### 3.2. Oil–Water Separation Tests

**Figure 6:**
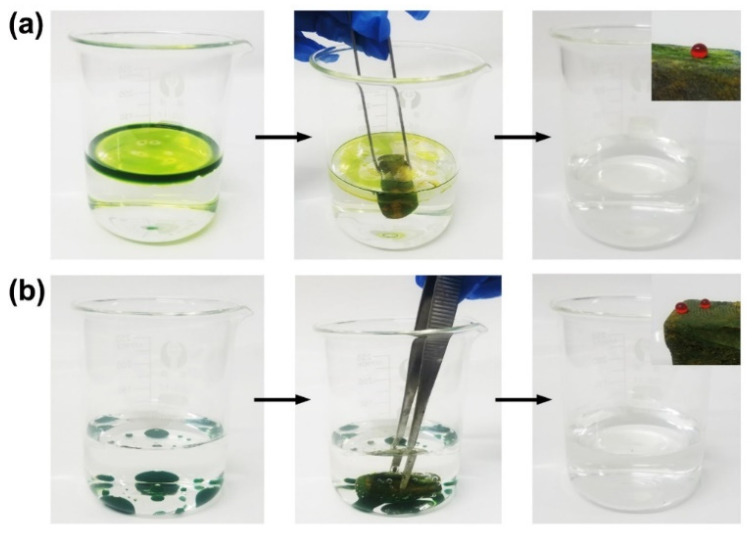
Photographs of the oil–water separation tests. (**a**) The absorption of the gasoline (dyed with chrome oxide green) from the gasoline–water mixture, (**b**) the absorption of chloroform (dyed with chrome oxide green) from the chloroform–water mixture.

To study the oil–water separation ability of the superhydrophobic cuttlebone, a simple oil–water separation test was carried out (Figure 6). First, a gasoline–water mixture and a chloroform–water mixture were prepared, wherein gasoline and chloroform were colored with chromium oxide green to enhance the contrast between the oil layer and the water layer. When a superhydrophobic cuttlebone sample was placed in a beaker containing the gasoline–water mixture, the top oil layer was fully absorbed within 30 s (Figure 6a). Similarly, when a superhydrophobic cuttlebone sample was pushed to the bottom of a beaker containing the chloroform–water mixture, the green chloroform layer was gradually absorbed by the superhydrophobic cuttlebone sample, as shown in Figure 6b. These results indicate that the superhydrophobic cuttlebone has excellent oil–water separation ability.

To investigate the durability of the superhydrophobic coating, the static water contact angles of the superhydrophobic cuttlebone samples after the gasoline–water and chloroform–water tests were measured, and they were 150° and 151°, respectively (Table 1). The result shows that the superhydrophobic cuttlebone samples still maintain excellent superhydrophobicity after the oil–water separation tests, implying the high adhesion and stability of the interfaces between the polydopamine coating layer, the fluorinated silica micro/nanoparticles, and cuttlebone.

**Figure 7:**
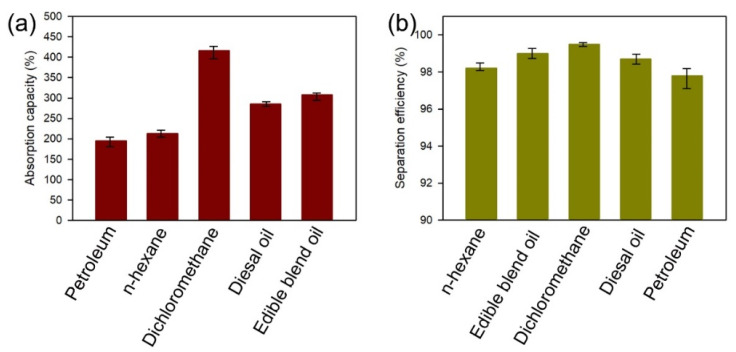
Absorption capacities (**a**) and oil–water separation efficiencies (**b**) of the superhydrophobic cuttlebone for various oil and organic solvents.

To investigate the oil absorption capacity of the superhydrophobic cuttlebone, superhydrophobic cuttlebone samples were first immersed in petroleum, n-hexane, dichloromethane, diesel, and cooking oil for about 10 min. After removing the excess oil on the surface, the samples were weighed, and the oil absorption capacities of the superhydrophobic cuttlebone in different organic liquids were calculated using Equation (1). The superhydrophobic cuttlebone shows excellent absorption capacity ranging from 195% to 416%, as shown in Figure 7a. To further explore the oil–water separation efficiency of the superhydrophobic cuttlebone for the oils of different viscosities and densities, various oil–water mixtures were prepared using petroleum, n-hexane, dichloromethane, diesel, and cooking oil as the oil layer. In all four tests, the oil layer was absorbed by the superhydrophobic cuttlebone within 30 s. The oil–water separation efficiencies based on Equation (2) are all above 97% (Figure 7b).

## 4. Conclusions

In this study, an efficient, convenient, and sustainable superhydrophobic material was developed using porous biomass cuttlebone as the scaffold and a layer of polydopamine as the adhesion layer between the cuttlebone and the superhydrophobic coating. The adhesion layer was obtained via the self-polymerization of dopamine on the surface of cuttlebone under a weak alkaline condition. The in situ grown silica micro/nanoparticles on the adhesion layer provide the anchoring spots for grafting the fluorinated hydrocarbon and a rough topography to realize superhydrophobicity. The static water contact angle of the superhydrophobic cuttlebone reaches 152°, and its oil contact angle is ~0°. The oil absorption tests and oil–water separation tests demonstrate a good superhydrophobicity of the superhydrophobic cuttlebone. Its oil adsorption capacity ranges from 195% to 416%, and its oil–water separation efficiency is over 97%. Besides, the oil absorption tests and oil–water separation tests have little influence on the superhydrophobicity of the superhydrophobic cuttlebone, showing the excellent durability of the superhydrophobic coating. The preparation of the superhydrophobic porous material based on a degradable and sustainable marine biomass waste, cuttlebone, has great potential for managing oil spills in oceans and rivers, opening a new avenue for transforming biomass waste into high-value functional materials.

## Figures and Tables

**Table 1:** Contact angle of superhydrophobic cuttlebone before and after oil absorption.

Samples	Contact Angle
Superhydrophobic cuttlebone	152°
After absorbing gasoline	150°
After absorbing chloroform	151°

## Data Availability

Not applicable.
